# *Staphylococcus aureus* infection disparities among Hispanics and non-Hispanics in Yuma, Arizona

**DOI:** 10.1017/ash.2024.390

**Published:** 2024-09-18

**Authors:** Talima Pearson, Sarah Kramer, David Panisello Yagüe, Emmanuel Nangkuu, Sarah Medina-Rodriguez, Colin Wood, Crystal Hepp, Ricky Camplain, Joseph Mihaljevic, Trudie Milner

**Affiliations:** 1 Pathogen & Microbiome Institute, Northern Arizona University, Flagstaff, AZ, USA; 2 Yuma Regional Medical Center, Yuma, AZ, USA; 3 School of Informatics, Computing, and Cyber Systems, Northern Arizona University, Flagstaff, AZ, USA; 4 Center for Health Equity Research, Northern Arizona University, Flagstaff, AZ, USA

## Abstract

*Staphylococcus aureus* infection patterns in Yuma, Arizona show a 2.25x higher infection rate in non-Hispanics. Males had higher infection rates in most age classes. These disparities in infection are mostly consistent with previously observed patterns in colonization, suggesting that sex and ethnicity do not differentially impact colonization and infection.

## Introduction

*Staphylococcus aureus* is a common cause of skin and soft tissue infections and also causes infections in the lungs, bones and joints, heart, and bloodstream.^1^ *S aureus* live in close association with humans as a commensal member of our microbial community, and infections are commonly due to autoinfection when *S. aureus* penetrate the outer layers of skin and mucosa.^1^

Comparing infection patterns across demographic categories can indicate which groups are at highest risk, allowing for the generation of causative hypotheses (eg, occupational, socioeconomic, social interactions, behaviors, or innate biological differences) to gain insights into underlying mechanisms of colonization, spread, and infection. For *S. aureus*, infection comparisons across sex and racial/ethnic groups in the U.S. have mostly focused on methicillin-resistant *S. aureus* (MRSA) infections and show a higher frequency in males, and black persons (compared to white persons).^2^ Reasons behind sex-based differences have not been identified, but disparities in invasive community-acquired MRSA cases can be explained mostly by socioeconomic factors.^3^

Here we describe *S. aureus* infection patterns at the Yuma Regional Medical Center (YRMC) in Yuma, Arizona. The YRMC catchment area includes all of Yuma County in Arizona and nearby communities in the Imperial Valley of California. This primarily Hispanic area on the United States/Mexico border has been the site of our recent work aimed at understanding community carriage of *S. aureus*.^4–6^ As carriage is a major risk factor for infection, the extent to which colonization and infection patterns mirror each other can shed light on how demographic factors might differentially influence colonization and infection. We are not aware of any studies describing infection in a single discrete population with known colonization patterns.

## Methods

### YRMC hospital account database

YRMC is a 406-bed hospital and the only major medical facility in the region. To determine the frequency of *S. aureus* infections, we parsed 1,323,446 hospital account records (a unique record created for each inpatient and outpatient hospital encounters resulting in a facility charge) created between 01/01/2015 and 08/01/2020. These dates were selected to bracket our study on community carriage of *S. aureus*.^6^ Of the 32,596 records that included a laboratory culture test, 2,045 included a positive culture test for *S. aureus.*

### *Age-adjusted* S. aureus *infection rates and hospital encounter rates for Hispanic and non-Hispanic males and females*

We used the direct age-adjustment method and the 2018 5-year population estimates for Yuma County in Arizona provided by the American Community Survey of the United States Census Bureau Yuma County census data as a standard population to calculate the age-adjusted *S. aureus* infection rates (based on positive culture tests) and hospital encounter rates (based on hospital account records) for the Hispanic and non-Hispanic White populations of both sexes. Calculations and underlying data can be found in Supplemental Table 1.

### Ethical oversight

The Northern Arizona Institutional Review Board determined that this project did not meet the definition of human subject research.

## Results

### *Population of* Yuma County, *Arizona*

**Figure 1.**
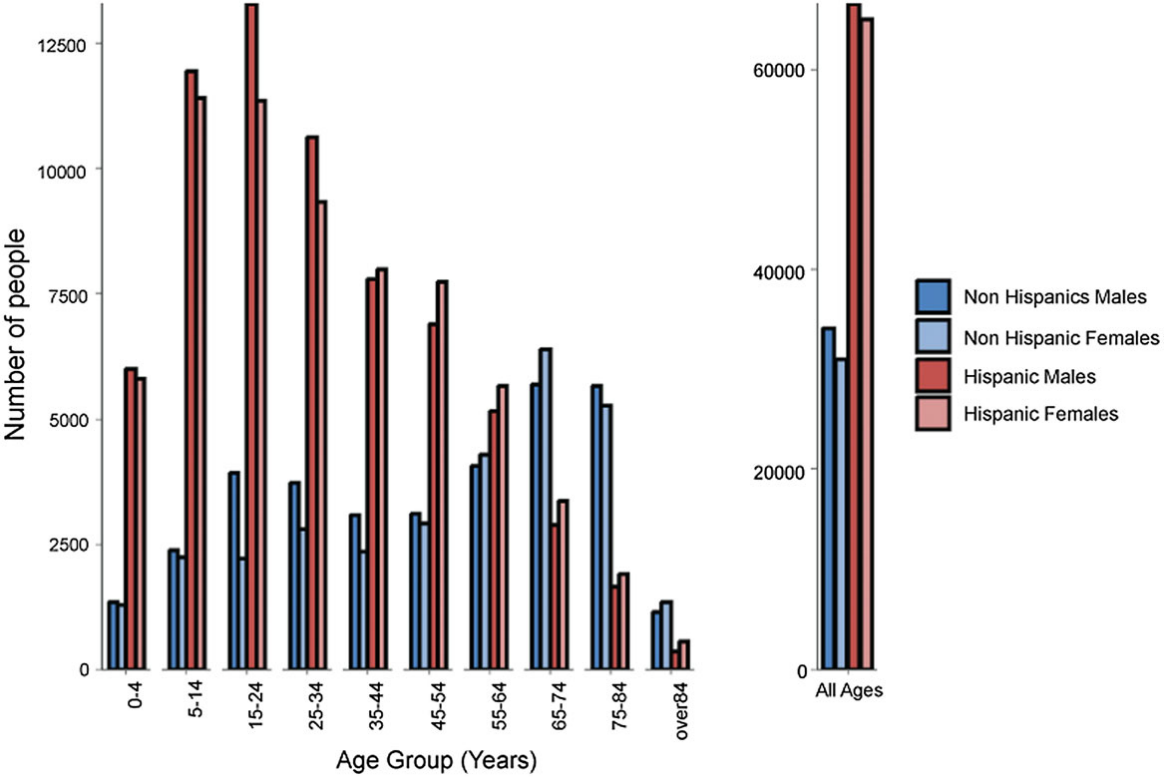
Demographics of Yuma County, AZ for non-Hispanic White and Hispanic males and females.

In Yuma, the Hispanic population is approximately twice the size of the non-Hispanic white population with an age distribution favoring the younger age classes (Figure 1). Yuma is a popular destination for retirees from other parts of North America, skewing the non-Hispanic white population distribution to older age classes.

### S. aureus *infections*

**Figure 2.**
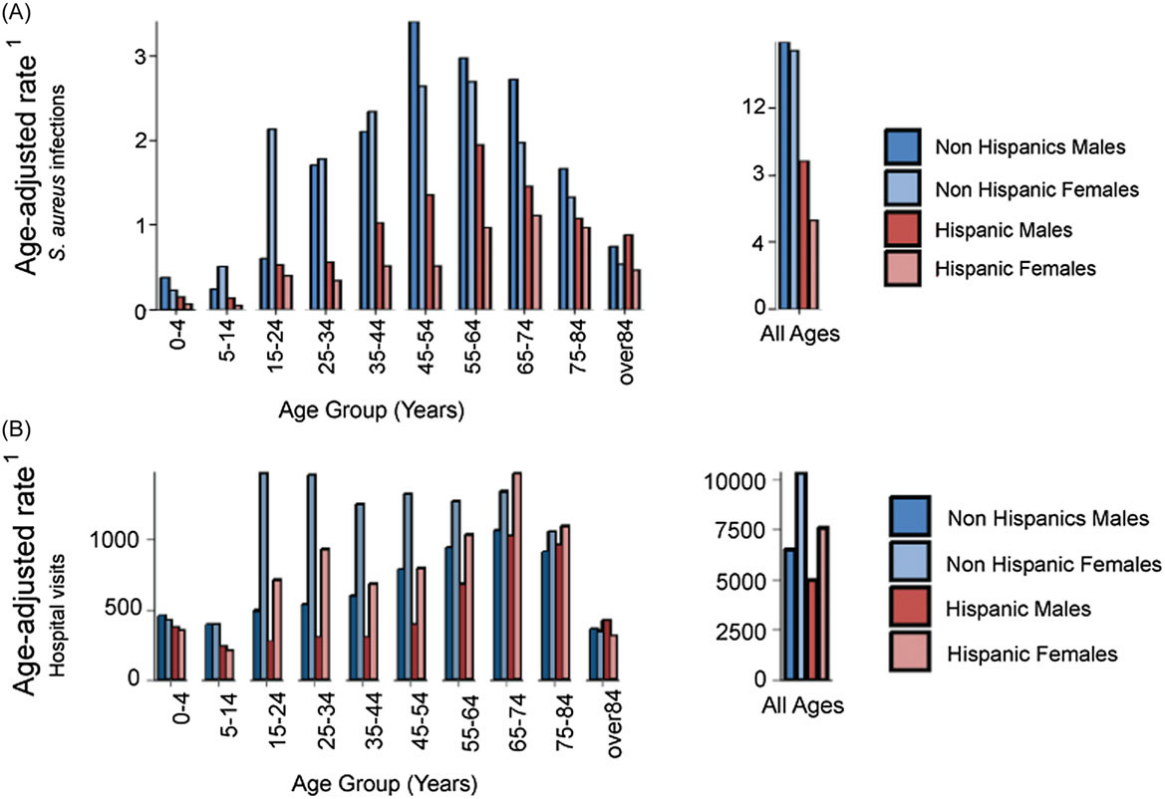
Age-adjusted rates of *S. aureus* infections (A) and hospital encounters (B) among Hispanic and non-Hispanic males and females. ^1^Rate per 1,000 people across the study time period.

Direct age adjustments for both ethnicities show that the total age-adjusted infection rate for non-Hispanics was 2.25x higher than for Hispanics, and higher in all but the oldest age class (Figure 2A). We also observed a sex-based disparity, however, this is only evident among Hispanics, where the infection rate in males is 1.69x higher than in females. For non-Hispanics, this pattern of higher rates in males did not occur in four of the younger age classes, explaining the lack of an overall sex-based difference (Figure 2A).

### Hospital encounter rates at YRMC

Hospital account records show that for each age class below 65, adjusted encounter rates (inpatient and outpatients) were greater for non-Hispanics (Figure 2B). Overall, the adjusted rate of YRMC encounters for non-Hispanics is 1.31x higher than for Hispanics. Overall, within each ethnicity group, and in most age classes, there is a sex-based disparity with female (especially of childbearing age and non-Hispanics) encounters more frequent than males (Figure 2B).

## Conclusions

Comparisons of age-adjusted *S. aureus* infection rates among the two main ethnicities show higher infection rates among non-Hispanics. All age classes of Hispanic males show higher infection rates than females, however, for non-Hispanics, some younger age classes show higher rate among females. Except for the youngest age class, these groups of non-Hispanic females have much higher hospital encounter rates which may result in a greater likelihood of *S. aureus* infection detection and thus offset the otherwise general trend of males having a higher infection rate than females. We did not collect identifying information and some patients will be linked to multiple records. While this may cause infection rates to be overestimated, the age-adjusted values presented here are not meaningful by themselves and should not be compared to rates from other studies. Our selected standard population is more likely to accurately reflect local demographics over the study period and thus will result in more precise sex and ethnic-based differences^7^ which can be compared across studies. While previous rate data on *S. aureus* are mostly limited to invasive MRSA infections, the sex-based disparity observed in this region is consistent with national trends.^2^ We are not aware of population-level studies comparing *S. aureus* infection rates across Hispanic and non-Hispanic ethnicities, however, infection rate disparities have been documented between other racial groups.^2^

Asymptomatic carriage can be an important marker for infection risk and prevalence. The degree to which carriage rates reflect infection rates can shed light on whether factors such as ethnicity, sex, and age differentially modify colonization and infection. As our previous work in this region shows little difference in carriage between age groups,^6^ it is possible that certain age groups are at higher risk of infection but not carriage, providing clues about underlying mechanisms of infection and colonization. In contrast, and consistent with this study, our previous work showed higher carriage rates in males and non-Hispanics.^6^ The data presented here for non-Hispanic females suggest that high infection rates may be linked to higher healthcare utilization, resulting in better detection. However, this does not explain the observed ethnic-based infection disparities which would be expected to disappear after the age of 65 when Medicare becomes available. Furthermore, Hispanic females have higher rates of healthcare utilization compared to Hispanic males, yet a sex-based infection disparity is evident. It is also possible that healthcare access and utilization does in fact increase detection in all demographic groups but causes a disproportionate impact in certain demographic groups.

In this population, where socioeconomic status does not explain colonization patterns,^4^ the similarities of infection and colonization patterns suggest that infection control will also not depend on socioeconomic status (SES)-based resources that can be mobilized to avoid preventative health risks. However, it is possible that certain types of infections may be linked to SES.^3,8–10^ Further exploring infection and colonization patterns may help unveil drivers and strategies to break the link between colonization and infection, leading to better protection and mitigation strategies.

## Supporting information

Pearson et al. supplementary material Pearson et al. supplementary material

## Supplementary material

For supplementary material accompanying this paper visit http://dx.doi.org/10.1017/ash.2024.390. click here to view supplementary material
